# Stroke Risk in Survivors of Head and Neck Cancer

**DOI:** 10.1001/jamanetworkopen.2023.54947

**Published:** 2024-02-13

**Authors:** Pui Lam Yip, Huili Zheng, Timothy Cheo, Teng Hwee Tan, Shing Fung Lee, Yiat Horng Leong, Lea Choung Wong, Jeremy Tey, Francis Ho, Yu Yang Soon

**Affiliations:** 1 Department of Radiation Oncology, National University Cancer Institute, National University Hospital, Singapore, Singapore; 2 Clinical Research Unit, Khoo Teck Puat Hospital, Singapore, Singapore

## Abstract

**Importance:**

Survivors of head and neck cancers (HNC) have increased risk of stroke. A comprehensive report using standardized methods is warranted to characterize the risk and to inform on survivorship strategy.

**Objective:**

To determine the stroke risk in subpopulations of survivors of HNC in Singapore.

**Design, Setting, and Participants:**

This national, registry-based, cross-sectional study aimed to estimate stroke risk in subgroups of the HNC population between January 2005 and December 2020. Participants were identified from the Singapore Cancer Registry, the Singapore Stroke Registry, and the Registry of Birth and Deaths using relevant *International Classification of Diseases, Ninth Revision, Clinical Modification* (*ICD-9-CM*) and *International Statistical Classification of Diseases and Related Health Problems, Tenth Revision, Australian Modification* (*ICD-10-AM*) codes. HNC subgroups were defined based on patient demographic, disease, and treatment factors. Data were analyzed from September 2022 to September 2023.

**Exposure:**

Diagnosis of HNC.

**Main Outcomes and Measures:**

Both ischemic and hemorrhagic stroke were studied. The age-standardized incidence rate ratio (SIRR) and age-standardized incidence rate difference (SIRD) were reported. The Singapore general population (approximately 4 million) served as the reference group for these estimations.

**Results:**

A total of 9803 survivors of HNC (median [IQR] age at diagnosis, 58 [49-68] years; 7166 [73.1%] male) were identified. The most common HNC subsites were nasopharynx (4680 individuals [47.7%]), larynx (1228 individuals [12.5%]), and tongue (1059 individuals [10.8%]). A total of 337 individuals (3.4%) developed stroke over a median (IQR) follow-up of 42.5 (15.0-94.5) months. The overall SIRR was 2.46 (95% CI, 2.21-2.74), and the overall SIRD was 4.11 (95% CI, 3.37-4.85) strokes per 1000 person-years (PY). The cumulative incidence of stroke was 3% at 5 years and 7% at 10 years after HNC diagnosis. The SIRR was highest among individuals diagnosed at younger than 40 years (SIRR, 30.55 [95% CI, 16.24-52.35]). All population subsets defined by age, sex, race and ethnicity, HNC subsites (except tongue), stage, histology, and treatment modalities had increased risk of stroke compared with the general population. The SIRR and SIRD were significantly higher among individuals who had a primary radiation treatment approach (SIRR, 3.01 [95% CI, 2.64-3.43]; SIRD, 5.12 [95% CI, 4.18-6.29] strokes per 1000 PY) compared with a primary surgery approach (SIRR, 1.64 [95% CI, 1.31-2.05]; SIRD, 1.84 [95% CI, 0.923.67] strokes per 1000 PY).

**Conclusions and Relevance:**

In this cross-sectional study of survivors of HNC, elevated stroke risks were observed across different age, subsites, and treatment modalities, underscoring the importance of early screening and intervention.

## Key Points

**Question:**

What are the risks of stroke in subpopulations of survivors of head and neck cancer (HNC)?

**Findings:**

In this cross-sectional study that included 9803 survivors of HNC and the entire Singapore population of 4 million, the age-standardized incidence of stroke among survivors of HNC was 2.5 times higher than that of the general population. The increased stroke risks were observed in most age groups, HNC subsites, stages, and treatment modalities.

**Meaning:**

These findings suggest that stroke prevention should be highlighted in HNC survivorship programs.

## Teaser

This cross-sectional study assesses stroke risk in survivors of head and neck cancers compared with the general population in Singapore.

## Introduction

Head and neck cancer (HNC) is a heterogeneous group of cancers that originate from the lip, oral cavity, pharynx, larynx, paranasal sinuses, and salivary glands.^1^ With advancement in surgery, radiotherapy (RT), and systemic treatments, the number of survivors of HNC has been steadily increasing.^2,3^

Importantly, several studies have reported an increased risk of stroke in patients treated for HNC^4,5,6,7,8,9,10^ (eTable 1 in Supplement 1). However, the precise estimation of stroke risk varied across these studies, primarily due to the difference in methods used. For example, some studies used geographically distant general populations as their reference group.^4,5^ In addition, some studies focused on the stroke risk in patients with HNC using patients treated with surgery alone as the comparator group.^8,9^ Moreover, different summary measures were used to quantify stroke risk in these studies. Some studies used incidence rate ratios,^4,5,7,10^ while some used hazard ratios derived from Cox proportional hazards regression model.^6,8,9,11^ Furthermore, most studies reported an increased risk in the overall HNC cohort, without providing detailed information about the risks in subpopulations. This heterogeneity among previous studies has impeded clinicians from effectively using the findings to provide targeted counselling for specific HNC subpopulations. To address these gaps, we conducted a cross-sectional study with the aim to determine the stroke risk in survivors of HNC across a comprehensive range of subgroups defined based on patient demographic, disease, and treatment factors, comparing them with the general population in Singapore.

## Methods

This cross-sectional study was approved by the National Healthcare Group Domain Specific Review Board and received a waiver of patient consent because this study used deidentified datasets. This report followed the Strengthening the Reporting of Observational Studies in Epidemiology (STROBE) reporting guideline for cross-sectional studies.

### Setting

Singapore is a country with population of 5.45 million as of June 2021, of whom approximately 4 million are Singapore citizens and permanent residents and the rest are foreigners (excluded from this study). More than three-quarters of the population is of Chinese ethnicity. The Singapore Department of Statistics releases Singapore’s residential population estimates annually.^12^

### Participants

All patients diagnosed with HNC from the Singapore Cancer Registry (SCR) between January 1, 2005, and December 31, 2020, were screened for eligibility. Patients with history of stroke (regardless of etiology) prior to HNC diagnosis were excluded from this study. For patients with multiple stroke episodes, only the first stroke after HNC diagnosis was considered as the outcome of interest. All eligible patients were followed up from date of HNC diagnosis to event of interest (stroke), death, or end of study period on December 31, 2020.

### Data Sources

This study analyzes the data that were retrospectively collected by the Singapore stroke, cancer, and death registries. The 3 registries capture data on Singapore citizens and permanent residents. The reference population was Singapore citizens and permanent residents. Details of the data capture by the 3 registries are in the eMethods in Supplement 1. HNC diagnoses were retrieved from the SCR with specific *International Classification of Diseases, Ninth Revision, Clinical Modification (ICD-9-CM) *and *International Statistical Classification of Diseases and Related Health Problems, Tenth Revision, Australian Modification (ICD-10-AM) *codes. Notification of cancer diagnoses to the SCR is mandated by law.^13^ The Singapore Stroke Registry (SSR) is notified of patients with stroke through the medical claims data from the Ministry of Health, the public hospitals inpatient discharge summaries, and the national death registry. The *ICD-9-CM* and *ICD-10-AM* codes used by SSR are listed in eMethods in Supplement 1. Data from the SSR were used to determine whether the patients with HNC developed stroke after HNC diagnosis and to determine the stroke incidence rate among the general population.^14^ Annual audits ensured data accuracy and interrater reliability of at least 95% for both cancer^15^ and stroke^16^ registries.

All deaths occurring in Singapore are mandated by law to be registered in the Registry of Birth and Deaths. Data from the SCR and SSR were merged with the death registry to obtain the survival status and person-years of HNC and all patients with stroke over the study period to estimate the risk of stroke among patients with HNC compared with the general population. The event of interest is the first stroke after the diagnosis of HNC.

### Subgroups

The subgroups of interest related to demographics include age at HNC diagnosis, race and ethnicity, and sex. Subgroups related to HNC include subsites, histology, and stage. Subgroups related to treatment include surgery, RT, chemotherapy, or combinations of these. All data were obtained from the SCR based on documentation in the medical records. Race and ethnicity were classified as Chinese, Indian, Malay, and others (eg, Arab, Eurasian, and several ethnic minorities). Race and ethnicity were assessed because of their known associations with stroke and various subsites of HNC.

### Statistical Analysis

We estimated the risk of stroke among the HNC cohort compared with the general population using the age-standardized incidence rate ratio (SIRR) and rate difference (SIRD).^17^ The SIRR was calculated as the observed number of strokes among patients with HNC divided by the expected number of strokes among patients with HNC assuming age-specific stroke incidence from the general population.

The IRR was standardized with 10-year age groups. We estimated the standard error of the SIRR by assuming that the observed number of strokes follows a Poisson distribution and using the Greenland and Robins formula.^18^ The SIRR was judged to be statistically significant if its 95% CI did not include 1.

The SIRD was calculated by the following formula: (observed number of strokes among patients with HNC − expected number of strokes among patients with HNC assuming age-specific stroke incidence from the general population) / number of person-years (PY) at risk while being alive among patients with HNC. The number of PY at risk represented the duration of exposure when patients with HNC were alive. The SIRD was judged to be statistically significant if its 95% CI did not include zero.

We used the Ederer II method^19^ to estimate the cumulative incidence of stroke among patients with HNC. We also plotted the observed and expected cumulative incidence of stroke among patients with HNC, accounting for death as a competing risk.

*P* values were 2-sided. All statistical analyses were performed with Stata version 13 (StataCorp). Data were analyzed from September 2022 to September 2023.

## Results

Among 10 023 patients diagnosed with HNC from January 2005 to December 2020, the study population included 9803 patients with HNC (median [IQR] age at diagnosis, 58 [49-68] years; 7166 [73.1%] male) (Table 1). Overall, there were 8444 Chinese patients (86.1%), 499 Indian patients (5.1%), 697 Malay patients (7.1%), and 163 patients (1.7%) identified as another race or ethnicity. The most common HNC subsites were nasopharynx (4680 patients [47.7%]), larynx (1228 patients [12.5%]), and tongue (1059 patients [10.8%]). Among patients with HNC who did not develop stroke, the median (IQR) age was 58 (49-68) years. Among those with HNC who developed stroke, the median (IQR) age was 61 (52-70) years. The cohort that developed stroke was older, more likely to be male, and more likely to have non–squamous cell carcinoma compared with the cohort that did not develop stroke (Table 1).

**Table 1.** Demographics and Clinical Characteristics of the Cohort

Characteristics	Individuals, No. (%)			*P* value
	Overall (N = 9803)	No subsequent stroke (n = 9466)	With subsequent stroke (n = 337)	
Age at diagnosis of HNC, median (IQR), y	58 (49-68)	58 (49-68)	61 (52-70)	<.001
Sex				
Female	2637 (26.9)	2577 (27.2)	60 (17.8)	<.001
Male	7166 (73.1)	6889 (72.8)	277 (82.2)	
Race				
Chinese	8444 (86.1)	8152 (86.1)	292 (86.6)	.25
Indian	499 (5.1)	481 (5.1)	18 (5.3)	
Malay	697 (7.1)	671 (7.1)	26 (7.7)	
Others^a^	163 (1.7)	162 (1.7)	1 (0.3)	
Clinical characteristics related to HNC				
Subsite				
Lip	20 (0.2)	19 (0.2)	1 (0.3)	.08
Tongue	1059 (10.8)	1040 (11.0)	19 (5.6)	
Salivary gland	646 (6.6)	628 (6.6)	18 (5.3)	
Mouth	732 (7.5)	705 (7.4)	27 (8.0)	
Oropharynx	528 (5.4)	508 (5.4)	20 (5.9)	
Nasopharynx	4680 (47.7)	4509 (47.6)	171 (50.7)	
Hypopharynx	374 (3.8)	364 (3.8)	10 (3.0)	
Ill-defined sites within lip, oral cavity, and pharynx	23 (0.2)	23 (0.2)	0 (0)	
Nasal cavity, middle ear, and accessory sinus	513 (5.2)	496 (5.2)	17 (5.0)	
Larynx	1228 (12.5)	1174 (12.4)	54 (16.0)	
AJCC stage				
I	1273 (14.5)	1220 (14.4)	53 (16.9)	.11
II	1393 (15.8)	1336 (15.7)	57 (18.1)	
III	1987 (22.6)	1910 (22.5)	77 (24.5)	
IV	4142 (47.1)	4015 (47.3)	127 (40.4)	
Not reported	1008	985	23	NA
T category				
1	2185 (26.1)	2108 (26.1)	77 (25.2)	.94
2	1967 (23.5)	1889 (23.4)	78 (25.5)	
3	1633 (19.5)	1573 (19.5)	60 (19.6)	
4	2583 (30.9)	2492 (30.9)	91 (29.7)	
Not reported	1435	1404	31	NA
N category				
0	3015 (36.2)	2891 (36.0)	124 (40.7)	.06
1	1693 (20.3)	1638 (20.4)	55 (18.0)	
2	2727 (32.7)	2622 (32.6)	105 (34.4)	
3	902 (10.8)	881 (11.0)	21 (6.9)	
Not reported	1466	1434	32	NA
M category				
0	7562 (93.7)	7281 (93.6)	281 (96.2)	.07
1	512 (6.3)	501 (6.4)	11 (3.8)	
Not reported	1729	1684	45	NA
Squamous cell carcinoma	4899 (50.0)	4754 (50.2)	145 (43.0)	.009
Treatment within 6 mo of HNC diagnosis				
No treatment received	1361 (13.9)	1332 (14.1)	29 (8.6)	<.001
Surgery only	1264 (12.9)	1226 (13.0)	38 (11.3)	
Surgery and RT (no chemotherapy)	995 (10.1)	963 (10.2)	32 (9.5)	
Surgery and chemotherapy (no RT)	40 (0.4)	40 (0.4)	0 (0)	
Surgery and chemotherapy and RT				
Concurrent^b^	408 (4.2)	401 (4.2)	7 (2.1)	
Sequential^c^	58 (0.6)	57 (0.6)	1 (0.3)	
RT only	1996 (20.4)	1895 (20.0)	101 (30.0)	
Chemotherapy only	422 (4.3)	410 (4.3)	12 (3.6)	
RT and chemotherapy (no surgery)				
Concurrent^b^	2302 (23.5)	2216 (23.4)	86 (25.5)	
Sequential^c^	957 (9.8)	926 (9.8)	31 (9.2)	

Abbreviations: AJCC, American Joint Committee on Cancer; HNC, head and neck cancer; NA, not applicable; RT, radiotherapy.

^a^ Includes Eurasian, Arab, and several ethnic minorities.

^b^ Concurrent refers to start of RT and chemotherapy were no more than 2 weeks apart.

^c^ Sequential refers to start of RT and chemotherapy were more than 2 weeks apart.

### Outcome Data

The median (IQR) follow-up was 42.5 (15.0-94.5) months. A total of 337 patients (3.4%) developed a stroke after the diagnosis of HNC (Table 2). The risk of stroke was significantly higher among patients with HNC compared with the general population (SIRR, 2.46 [95% CI, 2.21-2.74]; SIRD, 4.11 [95% CI, 3.37-4.85] strokes per 1000 PY) (Table 3).

**Table 2.** Clinical Characteristics of the First Stroke After HNC Diagnosis

Characteristics	First stroke after HNC diagnosis, No. (%) (n = 337)
Time to first stroke, median (IQR), mo	55 (23-94)
Etiology	
Ischemic	291 (86.3)
Hemorrhagic	43 (12.8)
Unknown	3 (0.9)
Time to death after stroke, median (IQR), mo	5 (1-22)
Death within 30 d after stroke	48 (14.2)

Abbreviation: HNC, head and neck cancer.

**Table 3.** SIRR and SIRD of Stroke Defined by Patient Characteristics and Disease Factors

Characteristics	Survivors, No.	Stroke, No.		SIRR (95% CI)	SIRD (95% CI) per 1000 PY
		Observed	Expected		
Overall	9803	337	137	2.46 (2.21-2.74)	4.11 (3.37-4.85)
Within first 5 y from diagnosis of cancer	5905	178	97	1.84 (1.59-2.14)	2.65 (1.80-3.50)
After 5 y from diagnosis of cancer	3898	159	41	3.92 (3.36-4.58)	6.64 (5.26-8.03)
Age at diagnosis of HNC, y					
<40	839	13	1	30.55 (16.24-52.35)	2.16 (0.95-3.37)
40-49	1712	47	8	5.84 (4.29-7.78)	3.44 (2.25-4.63)
50-59	2750	96	31	3.13 (2.53-3.82)	4.25 (3.00-5.49)
60-69	2399	94	40	2.38 (1.92-2.91)	5.55 (3.62-7.49)
70-79	1459	58	38	1.53 (1.16-1.98)	4.16 (1.06-7.26)
≥80	644	29	21	1.41 (0.94-2.03)	6.14 (−1.49-13.77)
Sex					
Male	7166	277	121	2.28 (2.03-2.57)	4.58 (3.62-5.54)
Female	2637	60	31	1.97 (1.53-2.54)	2.02 (0.98-3.06)
Race and ethnicity					
Chinese	8444	292	112	2.60 (2.31-2.91)	4.21 (3.42-4.99)
Indian	499	18	7	2.52 (1.59-4.01)	5.05 (1.18-8.92)
Malay	697	26	11	2.31 (1.57-3.39)	4.81 (1.55-8.08)
Others^a^	163	1	NA	NA	NA
Subsite					
Tongue	1059	19	15	1.27 (0.81-1.99)	0.92 (−1.01-2.84)
Salivary gland	646	18	10	1.88 (1.18-2.98)	2.34 (0.03-4.64)
Mouth	732	27	12	2.26 (1.55-3.30)	5.10 (1.65-8.56)
Oropharynx	528	20	7	2.83 (1.82-4.38)	6.32 (2.04-10.61)
Nasopharynx	4680	171	54	3.19 (2.75-3.71)	4.41 (3.45-5.38)
Hypopharynx	374	10	4	2.33 (1.25-4.33)	6.31 (−0.55-13.18)
Nasal cavity, middle ear, and accessory sinus	513	17	7	2.52 (1.56-4.05)	4.62 (0.98-8.27)
Larynx	1228	54	28	1.91 (1.46-2.49)	4.55 (2.00-7.09)
AJCC stage					
I	1273	53	27	1.98 (1.51-2.59)	3.23 (1.47-5.00)
II	1393	57	24	2.39 (1.84-3.10)	3.74 (2.07-5.42)
III	1987	77	30	2.59 (2.08-3.24)	4.03 (2.57-5.50)
IV	4142	127	45	2.83 (2.38-3.37)	5.50 (4.02-6.98)
Histology					
Squamous cell carcinoma	4899	145	73	1.99 (1.69-2.34)	3.86 (2.60-5.12)
Nonsquamous cell carcinoma	4904	192	64	2.99 (2.59-3.44)	4.27 (3.37-5.18)

Abbreviations: AJCC, American Joint Committee on Cancer; HNC, head and neck cancer; NA, not available (due to insufficient events); PY, person-years; SIRD, standardized incidence rate difference; SIRR, standardized incidence rate ratio.

^a^ Includes Eurasian, Arab, and several ethnic minorities.

The cumulative incidence of stroke was estimated at 3% at 5 years and 7% at 10 years after HNC diagnosis^19^ (Figure). The risk of stroke was significantly higher among patients at 5 years after HNC diagnosis compared with within 5 years (SIRR, 3.92 [95% CI, 3.36-4.58] vs SIRR, 1.84 [95% CI, 1.59-2.14]; SIRD, 6.64 [95% CI, 5.26-8.03] strokes per 1000 PY vs SIRD, 2.65 [95% CI, 1.80-3.50 strokes per 1000 PY) (Table 3).

**Figure.**
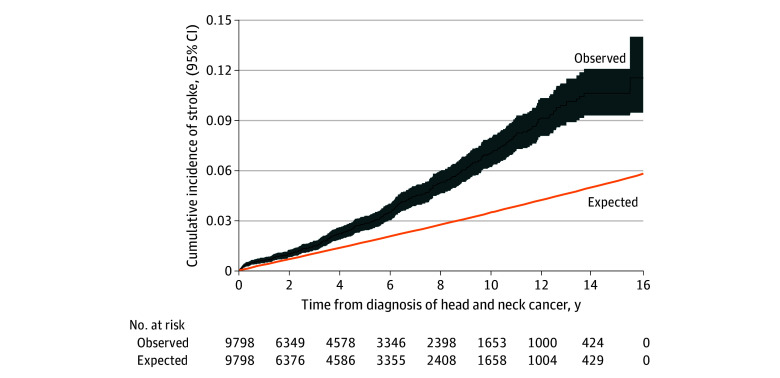
Observed and Expected Cumulative Incidence of Stroke in All Patients With Head and Neck Cancer Five patients had a stroke on the day of HNC diagnosis.

### Subgroups Defined by Demographics

The increased risk of stroke was evident across all age groups younger than 80 years (Table 3). The SIRR was highest among patients who were diagnosed with HNC when they were younger than 40 years (SIRR, 30.55 [95% CI, 16.24-52.35]). SIRR values decreased with increasing age at the diagnosis of HNC, whereas SIRD values showed an increasing trend (Table 3).

All population subsets defined by sex and race and ethnicity demonstrated an increased risk of stroke (Table 3). Furthermore, the SIRD for male patients was higher than for female patients (SIRD, 4.58 [95% CI, 3.62-5.54] strokes per 1000 PY vs 2.02 [95% CI, 0.98-3.06] strokes per 1000 PY).

### Subgroups Defined by Disease-Related Factors

Most population subsets defined by primary sites of disease, including salivary gland, mouth, oropharynx, nasopharynx, hypopharynx, and larynx, had significantly increased risk of stroke compared with the general population (Table 3). All the population subsets defined by American Joint Committee on Cancer (AJCC) stage and disease histology had significantly increased risk of stroke (Table 3). The SIRR of stroke for patients with nonsquamous histology was higher than for patients with squamous histology (SIRR, 2.99 [95% CI, 2.59-3.44] vs SIRR, 1.99 [95% CI, 1.69-2.34]). Characteristics of patients with squamous and nonsquamous histology are compared in eTable 2 in Supplement 1.

### Subgroups Defined by Treatment-Related Factors

The treatments received by patients within 6 months from HNC diagnosis are listed in Table 1. Most patients underwent concurrent chemoradiation (2302 patients [23.5%]) followed by RT alone (1996 patients [20.4%]). In addition, 1361 patients (13.9%) did not receive any treatment. Most population subsets defined by treatment modalities had significant increases in risk of stroke (Table 4). Notably, patients who did not receive any surgery, RT, or chemotherapy had significantly increased risk of stroke (SIRR, 2.18 [95% CI, 1.52-3.14]; SIRD, 3.23 [95% CI, 1.06-5.40] strokes per 1000 PY).

**Table 4.** SIRR and SIRD of Stroke Defined by Treatment Modalities

Treatment within 6 mo from HNC diagnosis	Survivors	Stroke, No.		SIRR (95% CI)	SIRD (95% CI) per 1000 PY
		Observed	Expected		
No treatment received	1361	29	13	2.18 (1.52-3.14)	3.23 (1.06-5.40)
Surgery only	1264	38	23	1.63 (1.18-2.24)	2.18 (0.38-3.97)
Surgery and RT	995	32	19	1.70 (1.20-2.40)	2.59 (0.41-4.77)
Surgery and chemotherapy	40	0	NA	NA	NA
Surgery, RT, and chemotherapy	466	8	6	1.44 (0.72-2.88)	1.13 (−1.43-3.70)
RT only	1996	101	36	2.80 (2.31-3.41)	6.00 (4.18-7.82)
Chemotherapy only	422	12	3	3.95 (2.25-6.96)	7.58 (1.84-13.32)
RT and chemotherapy	3259	117	37	3.20 (2.67-3.83)	4.56 (3.36-5.77)
Primary radiation treatment approach^a^	5255	208	73	3.01 (2.64-3.43)	5.12 (4.18-6.29)
Primary surgery approach^b^	2765	78	48	1.64 (1.31-2.05)	1.84 (0.92-3.67)

Abbreviations: HNC, head and neck cancer; NA, not available (due to insufficient events); PY, person-years; RT, radiotherapy; SIRD, standardized incidence rate difference; SIRR, standardized incidence rate ratio.

^a^ Primary RT treatment approach was defined as patients having RT only or RT and chemotherapy within 6 months from HNC diagnosis.

^b^ Primary surgery approach was defined as patients having surgery only, surgery and RT, surgery and chemotherapy, or surgery, RT, and chemotherapy within 6 months from HNC diagnosis.

Patients who had primary radiation treatment approach (defined as having RT only or RT and chemotherapy within 6 months from HNC diagnosis) had higher risk of stroke compared with those who had primary surgery approach (defined as having surgery only, surgery and RT, surgery and chemotherapy, or surgery, RT, and chemotherapy within 6 months of HNC diagnosis) (SIRR, 3.01 [95% CI, 2.64-3.43] vs SIRR, 1.64 [95% CI, 1.31-2.05]; SIRD, 5.12 [95% CI, 4.18-6.29] strokes per 1000 PY vs SIRD, 1.84 [95% CI, 0.92-3.67] strokes per 1000 PY) (Table 4). Characteristics of patients treated with primary surgery and primary radiation are compared in eTable 3 in Supplement 1.

Furthermore, the SIRR and SIRD of stroke were significantly higher for patients who had RT only compared with those who had surgery only (SIRR, 2.80 [95% CI, 2.31-3.41] vs SIRR, 1.63 [95% CI, 1.18-2.24]; SIRD, 6.00 [95% CI, 4.18-7.82] strokes per 1000 PY vs SIRD, 2.18 [95% CI, 0.38-3.97] strokes per 1000 PY) (Table 4).

## Discussion

In this registry-based cross-sectional study, we analyzed and reported the risk of stroke in different subgroups of survivors of HNC in a standardized and comprehensive manner. We used the age-standardized SIRR and SIRD, which compare the observed stroke events in HNC against the expected events. In contrast to other reports,^6,9,10^ we captured both ischemic and hemorrhagic stroke events from a national stroke registry. SIRR represents the relative risk, which is significantly influenced by the baseline rate in the reference population. Conversely, SIRD reflects the absolute difference in risks in terms of the excess number of strokes per 1000 PY, which is directly interpretable in terms of health outcomes among survivors. These 2 metrics should complement each other in revealing distinct risk profiles.

We observed that the standardized stroke risk in survivors of HNC was 2.46 times higher than the general population, with an excess of 4.11 strokes per 1000 PY. Stroke was prevalent among survivors of HNC, with an estimated cumulative incidence of 7% at 10 years after diagnosis.

A study by Chu et al^7^ approached the same clinical questions using a matched cohort, finding that stroke incidence was 1.44-fold higher in survivors of HNC. A study by Kim et al^11^ reported an IRD of 5.88 strokes per 1000 PY with propensity-score matching. A study by Yeh et al^10^ used a method similar to ours and reported an SIRR of 1.37 for ischemic stroke. However, their study did not report on SIRD nor detail the stroke risks in different subpopulations within the HNC cohort. Furthermore, nasopharyngeal carcinoma was excluded from their main analysis. We also note that the cohorts in the studies by Yeh et al^10^ and Kim et al^11^ predominately represented oral subsites (approximately 95% and 72%, respectively). In comparison, our cohort comprised of 48% nasopharyngeal, 18% oral cavity, and 13% laryngeal tumors. These studies highlighted the varying prevalence of HNC subsites across different geographical regions. Additionally, there is a global trend toward increasing incidence of lip and oral cavity cancers in low-middle sociodemographic index countries and increasing incidence of pharyngeal cancer in high sociodemographic index countries.^20^ An overall representation of stroke risk across all HNC subpopulation falls short of understanding the entire spectrum of the disease. Our report has the strength of providing a detailed account of SIRR and SIRD in different HNC subsites in a large population cohort, which would be of relevance for survivorship service planning and providing patient-specific counselling.

We reported a higher IRR of stroke compared with a recent cohort in Taiwan analyzed by Yeh et al.^10^ This discrepancy could be due to the overall higher incidence of ischemic stroke in Taiwan (70.5-81.7 strokes per 100 000 persons) than in Singapore (50.9-60.1 strokes per 100 000 persons).^21^ Also, our study captured both ischemic and hemorrhagic stroke, while the study by Yeh et al^10^ only focused on ischemic stroke. This observation also underscores a problem with the older studies^4,5^ that compared the stroke risk in patients with HNC with a geographically distant general population that may have a different underlying stroke risk. Furthermore, our estimates are particularly relevant in regions with similar reference stroke risk with Singapore.

Our data indicated that patients diagnosed at younger than 40 years had a striking 30.6-fold increased risk of developing stroke. This observation is in line with previous reports of high stroke risk in younger survivors of HNC.^4,7,8,22^ While the exact mechanism of this differential association of age with stroke is largely unknown, it is possible that complications from HNC treatments, including cisplatin-induced vascular toxic effects^23^ and radiation-induced carotid artery pathologies,^24,25,26,27^ pose a significant stroke risk in younger individuals. Moreover, it is widely believed that young age of exposure to cancer treatment is an important risk factor for later complications.^28^ Importantly, the point estimates of SIRR decreased and the SIRD increased with age. SIRR was higher in the younger population, reflecting the lower baseline stroke risk in age-matched individuals without established chronic diseases. SIRD was higher in older individuals, indicating a larger difference in stroke incidence rates between survivors of HNC and the general population.

We found that patients with nonsquamous histology had a higher SIRR than those with squamous histology. Within our cohort, the nonsquamous group was predominantly composed of nasopharynx cancers, which exhibited the highest SIRR among all subsites. Conversely, the squamous subgroup included nasopharynx, larynx, and tongue. In addition, more patients in the nonsquamous group received primary radiation approach. Our study also demonstrated that patients with HNC who were treated with primary radiation had significantly higher SIRR and SIRD compared with those treated with primary surgery. Many studies have postulated that radiation may induce carotid pathology,^24,25,26,27^ which could be linked to increased stroke. Nonetheless, our findings may also be influenced by various factors, including the underlying HNC subsites, stages, associated lifestyle factors, on top of the specific treatment modalities used. It may also reflect physicians’ bias to recommend radiation-based treatment for patients with more medical comorbidities, who are also at higher risk for stroke. Unfortunately, our dataset did not allow us to investigate the individual contributions of these factors to the observed outcomes.

Many studies on HNC stroke risks used Cox proportional hazard models to identify independent risk factors and quantity the respective risks while adjusting for other variables.^6,8,9,10,29^ This method has limitations, as it can only control for known covariates and relies on statistical assumptions. Indeed, HNC is a heterogeneous group of cancers, and the interplay between disease-, patient-, and treatment-related factors is complex. Interpreting the risk associated with an isolated variable while controlling for others is not intuitive. For example, it may not be sensible to interpret the adjusted hazard ratio for surgery after controlling for stage and subsite. In this study, we took a different approach, comparing stroke risk with the general population and identifying subpopulations of HNC that may require special stroke prevention. Although we observed elevated stroke risks irrespective of age, sex, race and ethnicity, subsite, stage, and treatment modality, more pronounced stroke risks were identified in certain subpopulations. First, SIRR and SIRD were notably higher in the 5 years following cancer diagnosis and in patients receiving primary radiation. Second, the highest point estimates of SIRR and SIRD were among those with nasopharynx, oropharynx, and mouth primary tumors. Third, younger populations exhibited much higher SIRR. These findings could inform resource allocation for stroke prevention and emphasize the importance of long-term follow-up.

Hypertension and diabetes have been shown to be associated with stroke in HNC.^30^ More importantly, a study of US veterans found that 47% of patients with HNC had uncontrolled cardiovascular risk factors at diagnosis, which were shown to be associated with incident stroke.^31^ Survivorship programs should focus on active screening and management of modifiable cerebrocardiovascular risk factors. However, there is a paucity of evidence on specific stroke prevention measures in HNC. Among them, some retrospective reports have suggested a protective role of statins.^32,33^ However, despite the associations demonstrated between carotid artery pathology and RT in HNC, regular carotid surveillance has yet to be proven to prevent stroke prospectively. Further research is necessary to investigate these strategies.

### Limitations

There are a few limitations in this study. First, the SCR does not have detailed information on comorbid conditions, lifestyle factors, and treatment information. Second, our outcome events were restricted to those captured by the SSR. These strokes were diagnosed at a hospital or resulted in death, which might underestimate the true incidence of stroke, including subclinical stroke^34^; however, our method can avoid misclassification. Third, human papillomavirus (HPV) status was not captured by the SCR during our study period. HPV-related HNC is of particular interest, as the prevalence is projected to increase globally, and HPV has been associated with higher stroke risk in HNC.^35^ Fourth, a longer follow-up will provide better estimates of stroke risk, since treatment-related complications often have a long latency period and can be compounded by chronic diseases and aging. Fifth, as the recruitment end date (latest HNC diagnosis date of included patients was December 31, 2020) and last follow-up date (censor date was December 31, 2020) are the same in this study, there might not have been enough time for stroke to occur, especially for patients with HNC diagnosed later during the study period. Hence, the risk of stroke in patients with HNC and their risk difference with the general population might be underestimated. Sixth, our study is a descriptive epidemiologic study,^36^ such that the data were not analyzed to make predictions or find causal inference. Despite these limitations, the use of robust and standardized statistical methods and access to dedicated national registries data had strengthened the reliability and validity of our results and emphasized the importance to recognize stroke risks in survivors of HNC.

## Conclusions

This cross-sectional study found that the incidence of stroke among survivors of HNC was approximately 2.5 times that of the general population in this cohort of individuals in Singapore. The increased risks of stroke were observed in most age groups, HNC subsites, stages, and treatment modalities. These findings highlight the importance of screening and early intervention.

## Notes

Supplement 1.
eMethods.
**eTable 1.** Selected Reports on Stoke Risk in Patients With Head and Neck Cancer **eTable 2.** Demographics and Clinical Characteristics of the Cohort by Squamous Cell vs Non–Squamous Cell Carcinoma **eTable 3.** Demographics and Clinical Characteristics of the Cohort by Treatment

Supplement 2.
Data Sharing Statement
