# Supplementary material for: Stroke Risk in Survivors of Head and Neck Cancer
Source: JAMA Netw Open. 2024 Feb 13;7(2):e2354947. doi: 10.1001/jamanetworkopen.2023.54947 (PMC10865145; doi:10.1001/jamanetworkopen.2023.54947)
Supplement: Supplement 1. — eMethods. eTable 1. Selected Reports on Stoke Risk in Patients With Head and Neck Cancer eTable 2. Demographics and Clinical Characteristics of the Cohort by Squamous Cell vs Non–Squamous Cell Carcinoma eTable 3. Demographics and Clinical Characteristics of the Cohort by Treatment [file jamanetwopen-e2354947-s001.pdf]

Supplemental Online Content

Yip PL, Zheng H, Cheo T, et al. Stroke risk in survivors of head and neck cancer. *JAMA Netw Open*. 2024;7(2):e2354947. doi:10.1001/jamanetworkopen.2023.54947

eMethods.

eTable 1. Selected Reports on Stoke Risk in Patients With Head and Neck Cancer

eTable 2. Demographics and Clinical Characteristics of the Cohort by Squamous Cell vs Non–Squamous Cell Carcinoma

eTable 3. Demographics and Clinical Characteristics of the Cohort by Treatment

This supplemental material has been provided by the authors to give readers additional information about their work.

© 2024 Yip PL et al. *JAMA Network Open.*

eMethods

This study analyzes the data that were retrospectively collected by the Singapore Stroke, Cancer, and Death registries. The three registries capture data on Singapore residents. The reference population in this study consisted of Singapore citizens and permanent residents, which comprised approximately 4 million individuals.

The (SCR) identifies cancer cases through notification by both public and private pathology laboratories and health care institutions, as well as the national Death Registry. Notification to the SCR of cancer cases is mandated by law [12]. The diagnosis of HNC is made based on the site of the primary lesion and histology, and all cases are coded for documentation. HNC falls under International Classification of Diseases, 9th Revision, Clinical Modification (ICD-9-CM) codes 140-149 (for years before 2012) and ICD-10 Australian Modification (ICD-10-AM) codes C00 to C14 (for year 2012 and after). Using these ICD codes, HNC cases were retrieved from the Cancer Registry for this study. Demographics and clinical characteristics pertaining to each HNC case are also captured by the Cancer Registry. Data was obtained via notifications received from (a) medical practitioners, (b) pathology laboratories, (c) haematology laboratories and departments, and (d) healthcare institutions. All notifications were corroborated with clinical medical records. Registry coordinators would review medical records to verify discrepancies in information and collect data to complete the registration of case records.[14]

The Singapore Stroke Registry is notified of the stroke cases through the medical claims data from the Ministry of Health, the public hospitals inpatient discharge summaries and the national Death Registry. The Stroke Registry employs the ICD-9-CM codes 430 to 437 (excluding 432.1 and 435) and ICD-10-AM codes I60 to I68

© 2024 Yip PL et al. *JAMA Network Open.*

(excluding I62.0 and I62.1) to identify the stroke cases diagnosed before 2012 and from 2012 onwards respectively. The etiology of stroke was made based on clinical assessment and confirmation by computed tomography or magnetic resonance imaging of the brain. Demographics and clinical characteristics pertaining to each stroke case are also captured by the Stroke Registry. Data from the Stroke Registry was used to determine if the HNC patients developed stroke after HNC diagnosis, and to determine the stroke incidence rate among the general population [13]. Annual audits ensured data accuracy and inter-rater reliability of at least 95% for both cancer [14] and stroke [15] registries.

All deaths occurring in Singapore are mandated by law to be registered in the Registry of Birth and Deaths. Data from the Cancer and Stroke Registries were merged with the Death Registry to obtain the survival status and person-years of HNC and all stroke patients over the study period to estimate the risk of stroke among HNC patients compared to the general population.

© 2024 Yip PL et al. *JAMA Network Open.*

Publications	Study Population	Comparator Population	Population size (Study population/ Comparator population)	Outcome	Summary Measure	Estimator	Estimate (95% CI)	Description of the risk of stroke
**Dorresteijn[4] 2002**	T1/2 Laryngeal Cancer and Parotid gland tumours (pleomorphic adenoma and parotid carcinoma) (age < 60 years old) treated with RT to head / neck from 1977 to 1998 at Netherlands Cancer Institute	General population in Oxfordshire, United Kingdom	367/ 105,000	Ischemic stroke	Incidence rate ratio	Incidence rate of stroke in the study population / Incidence rate of stroke in comparator population	5.6 (3.1–9.4)	The incidence of stroke in patients with early-stage laryngeal cancer and parotid tumors in the Netherlands was 5.6-fold that of the general population in Oxfordshire.
**Haynes[5] 2002**	Head and neck squamous cell carcinoma (age < 80 years old) treated with definitive RT to neck from 1987 to 2000 at University of Pennsylvania Medical Center, USA	General population in Stockholm, Sweden	413/ NA	Stroke and transient ischemic attack	Incidence rate ratio	Incidence rate of stroke in the study population / Adjusted incidence rate of stroke in the comparator population (adjusted for age, gender, smoking status)	2.09 (1.28–3.22)	The incidence of stroke in patients with head and neck squamous cell cancer treated with definitive radiation therapy to the neck in the United States was 2.09-fold that of the general population in Stockholm.
**Smith[6] 2008**	HNC (excluded laryngeal cancer) (age > 65 years old) treated with RT only from 1992 to 2002 in USA (SEER-Medicare)	1.HNC (excluded laryngeal cancer) (age > 65 years old) treated with surgery +RT only from 1992 to 2002 in USA (SEER-Medicare)	1983/ 2823	Cerebrovascular events (Stroke, carotid revascularization, and death due to stroke)	Hazard ratio	Multivariable Cox Proportional Hazards Regression Model	1.42 (1.14–1.77)	The hazards of cerebrovascular events (stroke, carotid revascularization, and death due to stroke) in patients with HNC, not including laryngeal cancer treated with RT only was 1.42-fold that of those with HNC, not including laryngeal cancer treated with surgery and RT adjusted for the confounding covariates.
		2. HNC (excluded laryngeal cancer) (age > 65 years old) treated with surgery from 1992 to 2002 in USA (SEER-Medicare)	1983/2056				1.5 (1.18–1.90)	The hazards of cerebrovascular events (stroke, carotid revascularization, death from stroke) in patients with HNC, not including laryngeal cancer treated with RT only was 1.5-fold that of those with HNC, not including laryngeal cancer treated with surgery alone adjusted for the confounding covariates.
**Chu[7] 2011**	Head Neck Cancer diagnosed from 2000 to 2002 in Taiwan (National Health Research Insitute)	Non-Cancer individuals diagnosed from 2000 to 2002 in Taiwan (National Health Research Insitute) with no prior stroke and matched for gender, age, hypertension and diabetes	13,390/53,517	Stroke (ischemic and haemorrhagic)	Incidence rate ratio	Incidence rate of stroke in the study population / Incidence rate of stroke in the comparator population	1.44 (1.33–1.57)	The incidence of stroke in patients with HNC diagnosed from 2000 to 2002 in Taiwan was 1.44-fold that of those without cancer and prior stroke in Taiwan, matched for gender, age, hypertension and diabetes.
					Hazard ratio	Cox Proportional Hazards Regression Model	1.52 (1.40–1.65)	The hazards of stroke in patients with HNC in Taiwan diagnosed from 2000 to 2002 was 1.52-fold higher than that of those without cancer and prior stroke in Taiwan, matched for gender, age, hypertension and diabetes.

© 2024 Yip PL et al. *JAMA Network Open.*

**Huang[8] 2011**	1.Head Neck Cancer treated with RT, chemo, chemo-RT from 2001 to 2002 in Taiwan (National Health Insurance Research Database)	Head Neck Cancer treated with surgery alone from 2001 to 2002 in Taiwan (National Health Insurance Research Database)	4391/2901	Stroke	Hazard Ratio	Multivariable Cox Proportional Hazards Regression Model	< 55 years old<br>1.76<br>(1.22–2.56)<br>>= 55 years old<br>0.74<br>(0.54–1.02)	Among those who under 55 years old, the hazards of stroke for patients with HNC treated with RT, chemo and chemo-RT was 1.76-fold that of those treated with surgery alone
	2. Head Neck Cancer treated with surgery with adjuvant therapy from 2001 to 2002 in Taiwan (National Health Insurance Research Database)		2880/2901				< 55 years old<br>0.80<br>(0.50–1.26)<br>>= 55 years old<br>1.00<br>(0.72–1.41)	
**Arthurs[9] 2016**	1.HNC diagnosed from 1990 to 2010 treated with RT only (age 35 to 75 years old) in Ontario, Canada	HNC diagnosed from 1990 to 2010 treated with surgery only (age 35 to 75 years old) in Ontario, Canada	4475/3120	Stroke and death from stroke	Hazard Ratio	Multivariable Cox Proportional Hazards Regression Model	1.70<br>(1.41–2.05)	The hazards of stroke for patients with HNC treated with RT only was 1.70-fold that of those treated with surgery only.
	2.HNC diagnosed from 1990 to 2010 treated with any RT only (age 35 to 75 years old) in Ontario, Canada		10949/3120				1.46<br>(1.23–1.73)	The hazard of stroke for patients with HNC treated with any RT was 1.46-fold that of those treated with surgery only.
**Yeh[10] 2022**	HNC, excluding nasopharyngeal carcinoma, diagnosed 2007 to 2016 (age 20 to 85 years old) in Taiwan (Taiwan Cancer Registry)	Random cohort of general population (age 20 to 85 years old) in Taiwan (Department of Household Registration)	41,266/<br>2,000,000	Ischemic stroke (National Health Insurance Research Database)	Age and sex standardized incidence rate ratio	Incidence rate of ischemic stroke in study population / incidence rate of ischemic stroke in general population, standardized by age and sex.	1.37<br>(1.30–1.44)	The incidence of stroke in patients with HNC was 1.37-fold that of the general population in Taiwan.
	HNC, excluding nasopharyngeal carcinoma, diagnosed 2007 to 2016 (age 20 to 85 years old) treated with any RT in Taiwan (Taiwan Cancer Registry)	HNC diagnosed 2007 to 2016 (age 20 to 85 years old) treated with surgery only in Taiwan (Taiwan Cancer Registry)	29,044/15,168		Hazard ratio	Multivariable Cox Proportional Hazards Regression Model	0.96<br>(0.83–1.10)	-
**This study**	HNC diagnosed 2005-2020 in Singapore (Singapore Cancer Registry)	Entire general population in Singapore	9803/<br>~4,000,000	Stroke (Ischemic and haemorrhagic)	Age standardized incidence rate ratio	Incidence rate of stroke in study population / incidence rate of stroke in general population, standardized by age	2.46<br>(2.21–2.74)	The incidence of stroke in patients with HNC was 2.46-fold that of the general population in Singapore.

Abbreviations: CI, confidence interval; HNC, head and neck cancer; RT, radiotherapy

eTable 1. Selected reports on stoke risk and in head and neck cancer patients.

© 2024 Yip PL et al. *JAMA Network Open.*

Characteristic	Non-SCC, N (%) (N=4904)	SCC, N (%) (N=4899)	P-value
**Demographics**			
Age at diagnosis of HNC, median (IQR), years	54 (45 – 63)	62 (54 – 71)	<0.001
Male	3375 (68.8)	3791 (77.4)	<0.001
Race			
Chinese	4358 (88.9)	4086 (83.4)	<0.001
Indian	122 (2.5)	377 (7.7)	
Malay	352 (7.2)	345 (7.0)	
Others [a]	72 (1.5)	91 (1.9)	
**Clinical characteristics related to HNC**			
Subsite			
Lip	11 (0.2)	9 (0.2)	<0.001
Tongue	79 (1.6)	980 (20.0)	
Salivary gland	613 (12.5)	33 (0.7)	
Mouth	192 (3.9)	540 (11.0)	
Oropharynx	127 (2.6)	401 (8.2)	
Nasopharynx	3485 (71.1)	1195 (24.4)	
Hypopharynx	30 (0.6)	344 (7.0)	
Ill-defined sites within lip oral cavity and pharynx	6 (0.1)	17 (0.3)	
Nasal cavity, middle ear and accessory sinus	295 (6.0)	218 (4.4)	
Larynx	66 (1.3)	1162 (23.7)	
AJCC stage			
1	512 (11.9)	761 (16.9)	<0.001
2	797 (18.6)	596 (13.2)	
3	1168 (27.3)	819 (18.2)	
4	1807 (42.2)	2335 (51.8)	
Not reported	620	388	
T category			
1	1020 (25.3)	1165 (26.9)	<0.001
2	1076 (26.7)	891 (20.5)	
3	851 (21.1)	782 (18.0)	
4	1085 (26.9)	1498 (34.5)	
Not reported	872	563	
N category			
0	1212 (30.2)	1803 (41.6)	<0.001
1	935 (23.3)	758 (15.5)	
2	1321 (33.0)	1406 (32.5)	
3	539 (13.4)	363 (8.4)	
Not reported	897	569	
M category			
0	3552 (92.0)	4010 (95.2)	<0.001
1	308 (8.0)	204 (4.8)	

© 2024 Yip PL et al. *JAMA Network Open.*

Not reported	1044	685	
**Treatment within 6 months from HNC diagnosis**			
No treatment received	681 (13.9)	680 (13.9)	<0.001
Surgery only	452 (9.2)	812 (16.6)	
Surgery and RT (no chemotherapy)	403 (8.2)	592 (12.1)	
Surgery and chemotherapy (no RT)	18 (0.4)	22 (0.4)	
Surgery and chemotherapy and RT			
Concurrent [b]	67 (1.4)	341 (7.0)	
Sequential [c]	22 (0.4)	36 (0.7)	
RT only	1027 (20.9)	969 (19.8)	
Chemotherapy only	252 (5.1)	170 (3.5)	
RT and chemotherapy (no surgery)			
Concurrent [b]	1325 (27.0)	977 (19.9)	
Sequential [c]	657 (13.4)	300 (6.1)	

Abbreviations: AJCC, American Joint Committee on Cancer; IQR, Interquartile range; HNC, head and neck cancer; RT, Radiotherapy; SCC, Squamous cell carcinoma
[a] Race “others” includes Eurasian, Arab and several ethnic minorities
[b] Concurrent refers to start of RT and chemotherapy were <=2 weeks apart
[c] Sequential refers to start of RT and chemotherapy were >2 weeks apart

eTable 2. Demographics and clinical characteristics of the cohort by (N=9803)

© 2024 Yip PL et al. *JAMA Network Open.*

Characteristic	Primary RT[b], N (%) (N=5255)	Primary surgery[c], N (%) (N=2765)	P-value
**Demographics**			
Age at diagnosis of HNC, median (IQR), years	57 (48 – 66)	60 (51 – 70)	<0.001
Male	4071 (77.5)	1751 (63.3)	<0.001
Race			
Chinese	4661 (88.7)	2239 (81.0)	<0.001
Indian	155 (2.9)	271 (9.8)	
Malay	366 (7.0)	200 (7.2)	
Others [a]	73 (1.4)	55 (2.0)	
**Clinical characteristics related to HNC**			
Subsite			
Lip	2 (0.04)	17 (0.6)	<0.001
Tongue	185 (3.5)	726 (26.3)	
Salivary gland	46 (0.9)	532 (19.2)	
Mouth	111 (2.1)	506 (18.3)	
Oropharynx	287 (5.5)	143 (5.2)	
Nasopharynx	3648 (69.4)	43 (1.6)	
Hypopharynx	214 (4.1)	82 (3.0)	
Ill-defined sites within lip oral cavity and pharynx	10 (0.2)	4 (0.1)	
Nasal cavity, middle ear and accessory sinus	139 (2.6)	281 (10.2)	
Larynx	613 (11.7)	431 (15.6)	
AJCC stage			
1	494 (9.7)	706 (28.2)	<0.001
2	899 (17.6)	396 (15.8)	
3	1488 (29.1)	342 (13.6)	
4	2230 (43.6)	1061 (42.4)	
Not reported	144	260	
T category			
1	1176 (23.7)	831 (34.0)	<0.001
2	1207 (24.3)	593 (24.3)	
3	1154 (23.3)	306 (12.5)	
4	1423 (28.7)	710 (29.1)	
Not reported	295	325	
N category			
0	1276 (25.7)	1504 (62.5)	<0.001
1	1224 (24.7)	305 (12.7)	
2	1831 (36.9)	514 (21.3)	
3	632 (12.7)	85 (3.5)	
Not reported	292	357	
M category			
0	4630 (95.8)	2301 (98.3)	<0.001

© 2024 Yip PL et al. *JAMA Network Open.*

1	204 (4.2)	40 (1.7)	
Not reported	421	424	
Squamous cell carcinoma	2246 (42.7)	1803 (65.2)	<0.001

Abbreviations: AJCC, American Joint Committee on Cancer; IQR, Interquartile range; HNC, head and neck cancer; RT, Radiotherapy
Primary
[a] Race “others” includes Eurasian, Arab and several ethnic minorities
[b] Primary RT includes RT only, or RT and chemotherapy within six months from HNC diagnosis
[c] Primary surgery includes surgery only, or surgery and RT, or surgery and chemotherapy, or surgery and RT and chemotherapy within six months from HNC diagnosis

eTable 3. Demographics and clinical characteristics of the cohort by treatment (N=8020)

© 2024 Yip PL et al. *JAMA Network Open.*
