# Supplementary material for: Stroke Risk in Survivors of Head and Neck Cancer
Source: JAMA Netw Open. 2024 Feb 13;7(2):e2354947. doi: 10.1001/jamanetworkopen.2023.54947 (PMC10865145; doi:10.1001/jamanetworkopen.2023.54947)
Supplement: Supplement 2. — Data Sharing Statement [file jamanetwopen-e2354947-s002.pdf]

Data Sharing Statement

Yip. Stroke Risk In Survivors of Head and Neck Cancer. *JAMA Netw Open*. Published February 06, 2024. doi:10.1001/jamanetworkopen.2023.54947

Data
Data available: Yes
Data types: Deidentified participant data
How to access data: The National Registry of Diseases, Singapore is the owner of these datasets. Access to these de-identified datasets is allowed if the Institutional Review Board and Ministry of Health, Singapore give their approval.
When available: With publication

Supporting Documents
Document types: None

Additional Information
Who can access the data: Researchers whose proposed use of the data has been approved
Types of analyses: Epidemiologic Cohort Study
Mechanisms of data availability: Access to these de-identified datasets is allowed if the Institutional Review Board and Ministry of Health, Singapore give their approval.
